# Surface Acoustic Wave Sensor with Pd/ZnO Bilayer Structure for Room Temperature Hydrogen Detection

**DOI:** 10.3390/s17071529

**Published:** 2017-06-29

**Authors:** Cristian Viespe, Dana Miu

**Affiliations:** National Institute for Laser, Plasma and Radiation Physics, Laser Department, Atomistilor # 409, 077125 Bucharest-Magurele, Romania; cristian.viespe@inflpr.ro

**Keywords:** SAW sensor, hydrogen sensor, bilayer, pulsed laser deposition, nanoporous film, Palladium, ZnO

## Abstract

A Surface Acoustic Wave (SAW) hydrogen sensor with a Pd/ZnO bilayer structure for room temperature sensing operation has been obtained by Pulsed Laser Deposition (PLD). The sensor structure combines a Pd layer with optimized porosity for maximizing mass effects, with the large acoustoelectric effect at the Pd/ZnO interface. The large acoustoelectric effect is due to the fact that ZnO has a surface conductivity which is highly sensitive to chemisorbed gases. The sensitivity of the sensor was determined for hydrogen concentrations between 0.2% and 2%. The limit of detection (LOD) of the bilayer sensor was about 4.5 times better than the single ZnO films and almost twice better than single Pd films.

## 1. Introduction

Hydrogen has many important applications such as alternative, clean energy sources, propulsion systems, and biomedical devices [1,2,3]. However, serious problems are implied by hydrogen use: in concentrations over 4.6% it is highly explosive [4], and over 4% it is extremely flammable [5]. The additional fact that hydrogen is colorless and odorless makes the need for sensors capable of detecting concentrations of 2% or less very important.

Sensors based on resistance, MOS-field effect transistors, or optical devices have been used for hydrogen detection, each having its advantages and disadvantages [6]. Surface acoustic wave (SAW) sensors have been widely used as hydrogen sensors due to advantages such as high sensitivity, fast response, reliability, and low cost [6,7,8]. Detection limits for H_2_ gas of 1250 to 2000 ppm (operating temperatures 80–120 °C) [9], 600 ppm (at 80 °C) [10] or as low as 200 ppm (at 175 °C) [11] using SAW sensors have been reported. In SAW sensors, the sensing mechanism is based on the perturbation of the surface acoustic wave propagation by mechanical and/or electrical effects. From a practical standpoint, the relevant effects for SAW sensing are changes in mass density and in electrical conductivity. In certain conditions, the changes in electrical conductivity can lead to a much stronger SAW sensor response than the changes in mass density [12]. This is because the acoustoelectric interactions between the electrical potential associated with surface acoustic wave propagation on a piezoelectric crystal and the mobile electric charges in the sensing film can lead to large modifications of the SAW attenuation and propagation velocity [13].

Bilayer SAW structures have been proven to increase the sensor sensitivity compared to single layer ones, and to decrease the detection limit, even in the case of room temperature operation [14,15]. By using bilayer films, the absolute value of the multilayer film conductivity can be adjusted to operate in a region where the acoustoelectric effect is efficient. Various material combinations such as Pd/WO_3_, ZnO/GaN, AlN/ZnO, or ZnO/SiO_2_ have been explored for SAW gas sensors based on this effect [9,16,17,18]. In the case of hydrogen sensing, an efficient acoustoelectric effect is desirable, since the mass effect for hydrogen is smaller than for heavier gases [9].

We propose a SAW bilayer structure for the detection of hydrogen consisting in Pd/ZnO, which, to our best knowledge, has not been investigated. Pd has excellent hydrogen absorption properties, having the highest hydrogen solubility of any element at atmospheric pressure as well as thermodynamically favored H_2_ dissociation [19]. The absorbing properties are improved even more in the case of nanoporous Pd layers, as we have shown in previous experiments [20]. ZnO is a wide band-gap semiconductor which is simple, reliable, low-cost, and easily mass-produced [21]. It is a piezoelectric material with a high coupling coefficient K, which is a measure of the conversion efficiency between electrical and acoustic energy [22]. When a semiconducting ZnO layer is deposited on a piezoelectric substrate, its mobile carriers couple to the electric field associated with SAW propagation along the piezoelectric substrate, leading to SAW attenuation and velocity change [16]. An important characteristic of ZnO for our case is the strong sensitivity of its surface conductivity to adsorbed species [23,24], which will increase the overall sensitivity of the bilayer sensor to hydrogen. It is worth mentioning that ST quartz substrates, which are used in our case, have a low temperature coefficient in comparison to other materials used for SAW substrates such as LiNbO_3_ or LiTaO_3_, and are therefore less sensitive to temperature variations [25]. We have therefore considered a bilayer structure consisting in a combination of a Pd layer with optimized porosity for maximizing mass effects, and a ZnO layer having an electron conductivity which is very sensitive to the exposure of the surface to hydrogen. Thus, both changes in mass density and in electrical conductivity contribute to the sensor response, leading to a SAW sensor with large hydrogen sensitivity.

## 2. Materials and Methods

### 2.1. Film Deposition and Characterization

**Figure 1:**
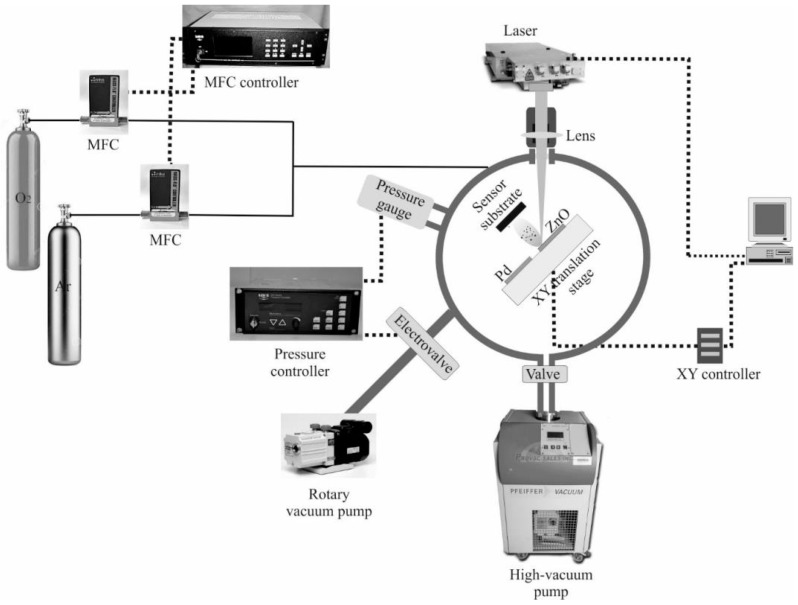
Experimental setup of sensitive layer deposition.

The sensitive layers of the SAW sensors were deposited onto ST-X quartz substrates by pulsed laser deposition (PLD). PLD was used because it presents a series of advantages in thin film deposition in general [17,26], and in particular has been proven to allow good control of sensitive film porosity, which is essential for SAW gas sensors [20]. The beam of a Nd:YVO_4_ laser is focused onto the surface of the target, leading to ablation of the target material and deposition onto a substrate placed at a distance of 35 mm in front of the target and parallel to its surface (Figure 1). The laser generates pulses of 10 ps duration, at a 10 kHz repetition rate, and a wavelength of 532 nm. The targets and substrate are placed in a vacuum chamber equipped with a system for the control of the gas pressure and flow. Mass flow meters are placed on the gas bottles and controlled by a mass flow controller, combined with a throttle valve placed on a rotary vane vacuum pump controlled by a pressure controller maintain the desired pressure in the ablation chamber and a controlled gas flow. Targets are placed on computer-controlled x-y tables which ensure continuous target movement during deposition, avoiding target erosion which leads to large film roughness. The system also permits deposition from multiple targets for multilayers. The laser energy density on the target can be modified by changing the distance between the focusing lens and the target.

**Figure 2:**
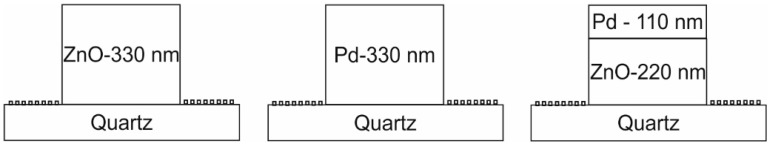
Schematic diagram of film structure.

In order to compare the sensing properties of single layer and bilayer SAW sensors, we have deposited a series of films all having a thickness of 330 nm (Figure 2), as measured using a QUANTA E = 50 keV Scanning Electron Microscope (SEM) and a Tokyo Seimitsu Surfcom 130 A profilometer. The bilayer film has a thinner metal layer (Pd) on top of the semiconducting layer (ZnO) since this is known to lead to a higher sensitivity [13,15]. In addition, the very good H_2_ absorption properties of Pd ensure penetration of the gas species to the ZnO layer. The Pd, ZnO, and Pd/ZnO thin films were directly deposited onto the quartz substrates at an average laser power of 0.7 W in a gas flow of 0.5 sccm. Before deposition, the chamber was evacuated to a base pressure of 10^−5^ Torr. In the case of Pd, films were obtained by ablation of a pure Pd target (99.95%) in an Ar atmosphere of 400 mTorr. The ZnO films were obtained by ablation of a ZnO target (99.95%) in O_2_ at 400 mTorr. All depositions were made at room temperature, and no post-deposition thermal treatments were used.

The morphology of the sensing films was studied by SEM. The crystalline structure of the films and the average size of the crystallites was determined by XRD, with a Panalytical X-ray diffractometer for films and powders.

### 2.2. Sensor Structure and Testing

**Figure 3:**
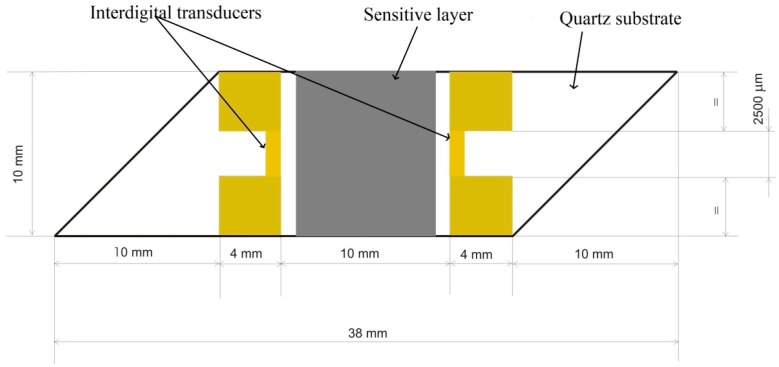
Scheme and dimension of SAW delay line.

The final value of the central frequency of the SAW device is circa 69.5 MHz; this is a decrease of about 300 kHz from the initial value, before the film deposition. The SAW sensors (delay line type), consisted of two port resonators with 50 electrodes pairs, with a periodicity of 11 µm (Figure 3). The periodicity of the IDTs was 45 µm and a 2500 µm wide acoustic aperture. The center-to-center distance between IDTs was 10 mm. The IDTs were obtained using standard photolithographic techniques, and consist in a 150-nm thick gold layer on top of a 10 nm chromium layer, the latter ensuring good adhesion to the quartz substrate [27,28,29,30]. The 10 × 38 mm^2^ quartz substrate was cut at a 45° angle, in order to reduce the effect of spurious SAW reflection from the edge of the piezoelectric substrate [31].

The loss signal in the oscillation circuit of the sensors was amplified (DHPVA-200 FEMTO amplifier), and the system frequency shift was analyzed (CNT-91 Pendulum counter analyzer and Time View III software). A network /spectrum /impedance analyzer (Agilent 4396B) which includes a transmission/reflection test kit (Agilent 87512 A/B) was used to optimize the inductor values and measure the signal attenuation and phase.

**Figure 4:**
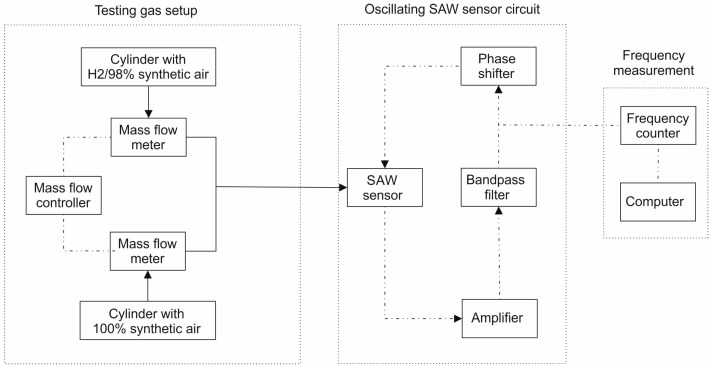
Experimental setup for SAW-sensor frequency shift measurements for hydrogen (H_2_) detection.

The performances of the sensors were determined at various hydrogen concentrations. Mass flow controllers (Figure 4) were used to combine the hydrogen gas mixture (2% H_2_/98% synthetic air) with pure synthetic air, thus obtaining different gas concentrations. The total gas flow rate was maintained at a constant value of 0.5 L/min in all cases. All sensors were measured at room temperature.

## 3. Results

### 3.1. Film Morphology and Structure

**Figure 5:**
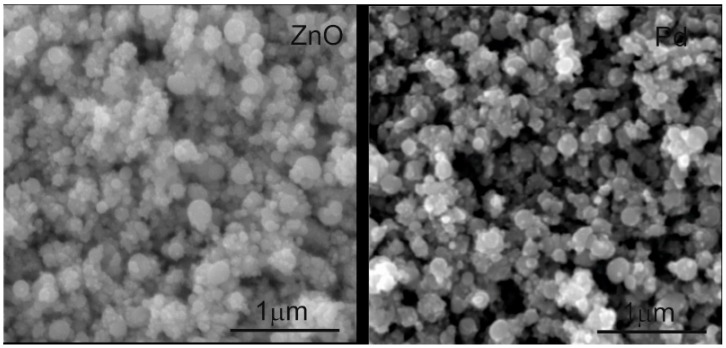
Scanning electron microscopy (SEM) images of the ZnO and Pd nanoporous films grown on the SAW sensors.

The 400 mTorr gas pressure used for laser ablation in our case leads to nanoporous films both in the case of ZnO and of Pd, as can be seen in the SEM images presented in Figure 5. Such relatively high laser deposition pressures are known to lead to films having a larger porosity [20]. This is due to collisions of the ablated species with the background gas, which lead to lowering of their kinetic energy and to cluster nucleation, producing films with a porous morphology [32,33]. Such nanoporous films lead to superior sensing properties due to rapid gas in/out diffusion and large surface area.

**Figure 6:**
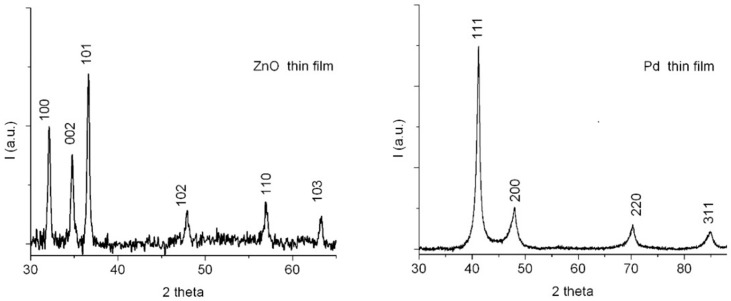
XRD spectra of the of ZnO and Pd films on quartz substrates.

Figure 6 presents the XRD results for the ZnO and Pd films. The patterns reveal the formation of pure polycrystalline compounds, a stoichiometric wurtzite ZnO phase, and a fcc Pd phase, respectively. For both thin films, the mean crystallite size values were in the nanometer range: 34 nm for ZnO and 13 nm for Pd. The deposition conditions which were used circumvent the known problem of potentially substantial Zn enrichment of the ablated target, which leads to nonstoichiometric PLD-deposited ZnO films [34], so that in our case the ZnO films are stoichiometric.

### 3.2. Sensor Properties

**Figure 7:**
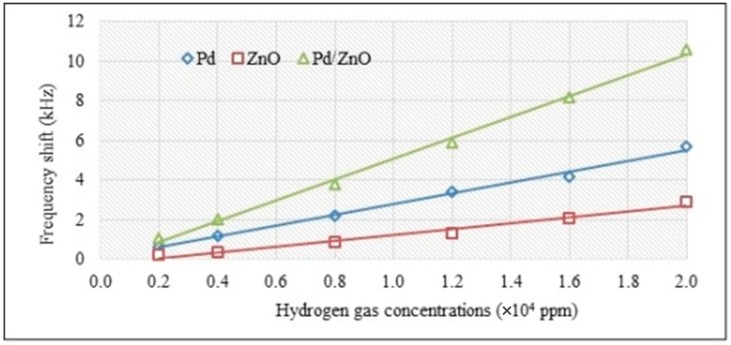
Frequency shift dependence of hydrogen concentration at RT.

The SAW sensor response was measured for the Pd, ZnO, and Pd/ZnO sensitive layers for hydrogen concentrations up to 2% (half of the safety limit for this gas). Figure 7 shows the response of the SAW sensor at different gas concentrations, at room temperature; each experimental point is the average result of five measurements. It was observed that the frequency shift Δf is proportional to the concentration for all sensors for a gas concentration between 2000 and 20000 ppm. These results are similar to those reported by Phan and Chung in the same concentration domain, for room temperature sensor operation [35].

Table 1 presents the sensitivities and detection limits for the sensitive layers of Pd, ZnO, and Pd/ZnO. The limit of detection (LOD) depends on the noise level, being defined as 3× noise level/sensitivity. The noise level was estimated at around 30 Hz for the all the films. The noise assessment was performed in air (without analyte) by measuring the frequency fluctuation over 10 min; it represents the maximum frequency deviation from the trend line (best-fit line). The sensitivity, defined as the frequency shift in Hz per unit analyte concentration in ppm, was determined from an average sensitivity value for a gas concentration between 0.2% and 2%. The response time (to reach 90% of maximum signal) for the sensors tested was between 12 and 16 s for these hydrogen concentrations.

The results in Table 1 indicate that the LOD of Pd/ZnO films was ~4.5 times better than the single ZnO films and almost 2-times better than the single Pd layers. In most cases, SAW sensors are heated in order to improve the hydrogen response [10,36]. Our results are obtained for room temperature operation, and are better than or comparable to those reported by other groups in such conditions [11,12,35,37].

## 4. Discussion and Conclusions

When a bilayer (metal/semiconductor) SAW sensor is exposed to air, oxygen is adsorbed and dissociates at the metal surface. The resulting atomic oxygen diffuses to the metal/semiconductor interface, where it captures electrons and increases the depletion layer width in the semiconductor. Subsequent exposure of the bilayer sensor to H_2_ similarly results in the formation of atomic hydrogen which diffuses to the interface, reacting with the previously generated oxygen species, and releasing electrons into the conduction band of the semiconductor. The increase in sheet electron conductivity will lead to a decrease in acoustic velocity, since the SAW wave is slowed down by the presence of carriers through the acoustoelectric effect [38,39].

Surface acoustic wave propagation is very sensitive to surface perturbations such as small changes in electrical conductivity. The acoustoelectric effect in the case of a single sensitive layer leads to a relative change of wave propagation velocity Δν given by

(1) $$\frac{\Delta f}{f_{0}}=\frac{\Delta \nu }{\nu_{0}}≅\frac{K^{2}}{2}\frac{\sigma_{s}^{2}}{\sigma_{s}^{2}+\nu_{0}C_{s}^{2}}$$

where Δf is the frequency shift, f_0_ the central frequency of the SAW device, ν_0_ the unperturbed SAW velocity, K the electromechanical coefficient, C_s_ = ε_0 +_ ε_p_ is the sum of the permittivity of the region above the film and the substrate, and σ_s_ is the surface conductivity of the sensing film [11,18]. In the case of bilayers, important additional parameters which determine velocity changes are the electrical conductivities of both layers and their thicknesses, since both film layers influence the SAW propagation through interaction between their mobile charges and the travelling electron potential φ of the SAW [17].

These mechanisms explain the improvement of the sensor properties in the case of the Pd/ZnO bilayer compared to the single Pd and ZnO layers. The hydrogen absorbtion and diffusion properties of Pd, combined with the excellent diffusion properties of the nanoporous Pd film, cause increased amounts of hydrogen to reach the Pd/ZnO interface. At this interface, the large sensitivity of ZnO surface conductivity to adsorbed species brings about increased hydrogen detection sensitivity. The sensor response of the bilayer Pd/ZnO thin film proposed by us thus combines both mass and acoustoelectric effects, leading to improved sensitivity to hydrogen. The mass effect of the gas on the sensing layer is known to always lead to a decrease of the center frequency of the SAW sensor. The variation of the electrical conductivity, on the other hand, can lead both to an increase and to a decrease of the center frequency [40]. In order for the combination of the mass and acoustoelectric effects to be efficient, the latter must lead to the same result, i.e., a decrease of the SAW center frequency. Otherwise, the two effects counteract each other and the combined response is smaller. As outlined above, this is the case for our sensor structure, since hydrogen is a reducing gas which reacts with the adsorbed oxygen species, releasing electrons into ZnO, increasing the surface conductivity and decreasing the center frequency. We consider that further optimization of the deposition conditions and sensor structure can lead to further improvement of the hydrogen sensing properties, and perhaps to applications for other gases.

In conclusion, a SAW bilayer structure consisting in porous Pd and ZnO layers has been obtained by PLD. The bilayer combines the excellent hydrogen absorbtion and dissociation properties with the sensitivity of surface electron conductivity of ZnO to adsorbed species. The SAW sensor based on this bilayer structure has an improved sensor response due to a combination of the mass and acoustoelectric effects appearing in the presence of hydrogen. The LOD of Pd/ZnO films is about 4.5 times better than the single ZnO films and almost twice better than the single Pd layers, reaching a value of 59 ppm. To our knowledge, it is for the first time that this material combination has been used for bilayers in hydrogen sensors based on surface acoustic waves.

Optimization of the bilayer structure can lead to further improvement of the bilayer Pd/ZnO sensor properties, and future research will be addressed in this direction. 

## Figures and Tables

**Table 1:** Sensitivity and limit of detection (LOD) of the sensors towards hydrogen (Δf = frequency shift; c = hydrogen concentration; n = noise level).

Coating Material	Sensitivity Δf/c (Hz/ppm)	LOD (3*n)/(Δf/c) (ppm)
Pd	0.29	105
ZnO	0.15	261
Pd/ZnO	0.51	59

## References

[1] Jacobson M.Z., Colella W.G., Golden D.M. (2005). Clearing the Air and Improving Health with Hydrogen Fuel-Cell Vehicles. Science.

[2] Bassam A.M., Phillips A.B., Turnock S.R., Wilson P.A. (2017). Development of a multi-scheme energy management strategy for a hybrid fuel cell driven passenger ship. Int. J. Hydrog. Energy.

[3] Grimes C.A., Ong K.G., Varghese O.K., Yang X., Mor G., Paulose M., Dickey E.C., Ruan C., Pishko M.V., Kendig J.W. (2003). A Sentinel Sensor Network for Hydrogen Sensing. Sensors.

[4] Boon-Brett L., Bousk J., Black G., Moretto P., Castello P., Hübert T., Banach U. (2010). Identifying performance gaps in hydrogen safety sensor technology for automotive and stationary applications. Int. J. Hydrog. Energy.

[5] Buttner W.J., Post M.B., Burgess R., Rivkin C. (2011). An overview of hydrogen safety sensors and requirements. Int. J. Hydrog. Energy.

[6] Hubert T., Boon-Brett L., Black G., Banach U. (2011). Hydrogen sensors—A review. Sens. Actuators B Chem..

[7] Cole M., Spulber I., Gardner J. (2015). Surface acoustic wave electronic tongue for robust analysis of sensory components. Sens. Actuators B Chem..

[8] Bhasker R.V., Nimal A.T., Parmar Y., Sharma M.U., Sreenivas K., Gupta V. (2010). Cross sensitivity and selectivity studies on ZnO surface acoustic wave ammonia sensor. Sens. Actuators B Chem..

[9] Jakubik W. (2009). Hydrogen gas-sensing with bilayer structures of WO_3_ and Pd in SAW and electric systems. Thin Solid Film.

[10] Ippolito S.J., Kandasamy S., Kalentar-Zadeh K., Wlodarski W. (2005). Layered SAW hydrogen sensor with modified tungsten trioxide selective layer. Sens. Actuators B Chem..

[11] Yang L., Yin C., Zhang Z., Zhou J., Xu H. (2017). The investigation of hydrogen gas sensing properties of SAW gas sensor based on palladium surface modified SnO_2_ thin film. Mater. Sci. Semicond. Process..

[12] Jakubik W., Urbanczyk M., Kochowski S., Bodzenta J. (2003). Palladium and phthalocynine bilayer films for hydrogen detection in a surface acoustic wave sensor system. Sens. Actuators B Chem..

[13] Jakubik W. (2014). Elementary theory of SAW gas sensor based on electrical conductivity changes in bi-layer nanostructures. Sens. Actuators B Chem..

[14] Jakubik W., Krzywiecki M., Maciak E., Urbańczyk M. (2012). Bi-layer nanostructures of CuPc and Pd for resistance-type and SAW-type hydrogen gas sensors. Sens. Actuators B Chem..

[15] Jakubik W., Powroznik P., Wrotniak J., Krzywiecki M. (2016). Theoretical analysis of acoustoelectrical sensitivity in SAW gas sensors with single and bi-layer structures. Sens. Actuators B Chem..

[16] Li R., Reyes P.I., Ragavendiran S., Shen H., Lu Y. (2015). Tunable surface acoustic wave device based on acoustoelectric interaction in ZnO/GaN heterostructures. Appl. Phys. Lett..

[17] Dutheil P.J., Orlianges C., Crunteanu A., Catherinot A., Champeaux C. (2015). AlN, ZnO thin films and AlN/ZnO or ZnO/AlN mulatilayer structures deposited by PLD for surface acoustic wave applications. Phys. Status Solidi A.

[18] Wang S.Y., Ma J.Y., Li Z.J., Su H.Q., Alkurd N.R., Zhou W.L., Wang L., Du B., Tang Y.L., Ao D.Y. (2015). Surface acoustic wave ammonia sensor based on ZnO/SiO_2_ composite film. J. Hazard. Mater..

[19] Noh J.S., Lee J.M., Lee W. (2011). Low-dimensional Palladium nanostructures for fast and reliable Hydrogen gas detection. Sensors.

[20] Viespe C., Grigoriu C. (2013). SAW sensor based on highly sensitive nanoporous palladium thin film for hydrogen detection. Microelectron. Eng..

[21] Janotti A., Van de Walle C.G. (2009). Fundamentals of zinc oxide as a semiconductor. Rep. Prog. Phys..

[22] Wu T.T., Wang W.S. (2004). An experimental study on the ZnO/sapphire layered surface acoustic wave device. J. Appl. Phys..

[23] Schmidt O., Kiesel P., Van de Walle C.G., Johnson N.M., Nause J., Döhler G.H. (2005). Effects of an electrically conducting layer on the zinc oxide surface. Jpn. J. Appl. Phys..

[24] Schmidt O., Geis A., Kiesel P., Van de Walle C.G., Johnson N.M., Bakin A., Waag A., Döhler G.H. (2006). Analysis of a conducting channel at the native zinc oxide surface. Superlattice. Microstruct..

[25] Ballantine D.S., White R.M., Martin S.I., Ricco A.J., Zellers E.T., Frye G.C., Wohltjen H. (1997). Acoustic Wave Sensors, Theory, Design and Physico-Chemical Applications.

[26] Chrisey D.B., Hubler G.K. (1994). Pulsed Laser Deposition of Thin Films.

[27] Marcu A., Viespe C. (2015). Laser-grown ZnO Nanowires for Room-temperature SAW-sensor Applications. Sens. Actuators B Chem..

[28] Viespe C. (2014). Surface Acoustic Wave Sensors based on Nanoporous Films for Hydrogen Detection. Mater. Appl. Sens. Transducer. Key Eng. Mater..

[29] Marcu A., Viespe C. (2016). Active surface geometrical control of noise in nanowire-SAW sensors. Sens. Actuators B Chem..

[30] Marcu A., Viespe C. (2017). Surface Acoustic Wave Sensors for Hydrogen and Deuterium Detection. Sensors.

[31] Campbell C. (1989). Surface Acoustic Wave Devices and THEIR Signal Processing Applications.

[32] Di Fonzo F., Tonini D., Li Bessi A., Casari C.S., Beghi M.G., Bottani C.E., Gastaldi D., Vena P., Contro R. (2008). Growth regimes in pulsed laser deposition of aluminum oxide thin films. Appl. Phys. A.

[33] Riabinina D., Irissou E., Le Drogoff B., Chaker M., Guay D. (2010). Influence of pressure on the Pt nanoparticle growth modes during pulsed laser ablation. J. Appl. Phys..

[34] Claeyssens F., Cheesman A., Henley S.J., Ashfold M.N. (2002). Studies of the plume accompanying pulsed ultraviolet laser ablation of zinc oxide. J. Appl. Phys..

[35] Phan D., Chung G. (2012). Surface acoustic wave hydrogen sensors based on ZnO nanoparticles incorporated with a Pt catalyst. Sens. Actuators B Chem..

[36] Rane S., Arbuj S., Rane S., Gosavi S. (2015). Hydrogen sensing characteristics of Pt-SnO_2_ nano-structured composite thin films. J. Mater. Sci. Mater. Electron..

[37] Jakubik W.P., Urbanczyk M., Mariak E., Pustelny T. (2009). Bilayer structures of NiO_x_ and Pd in Surface Acoustic Wave and electrical gas sensor systems. Acta Phys. Polonica A.

[38] Ricco A.J., Martin S.J., Zipperian T.E. (1985). Surface acoustic wave gas sensor based on film conductivity changes. Sens. Actuators.

[39] Fischer B.H., Malocha D.C. (2010). Study of the acoustoelectric effect for SAW sensors. IEEE Trans. Ultrason. Ferroelectr. Freq. Control.

[40] Fan H., Ge H., Zhang S., Zhang H., Zhu J. (2012). Optimization of sensitivity induced by surface conductivity and sorbed mass in SAW gas sensors. Sens. Actuators B Chem..

